# HDAC Inhibition Sensitize Hepatocellular Carcinoma to Lenvatinib via Suppressing AKT Activation

**DOI:** 10.7150/ijbs.93375

**Published:** 2024-05-19

**Authors:** Shuai Yan, Lu Chen, Hao Zhuang, Hui Yang, Yinmo Yang, Ning Zhang, Rong Liu

**Affiliations:** 1 Translational Cancer Research Center, Peking University First Hospital, Beijing 100034, China.; 2 Department of Hepatobiliary Tumor, Tianjin Medical University Cancer Institute and Hospital, Tianjin 300060, China.; 3 Department of Hepatic Biliary Pancreatic Surgery, Henan Cancer Hospital, Zhengzhou 450000, China.; 4 Department of General Surgery, Peking University First Hospital, Beijing 100034, China.; 5 International Cancer Institute, Peking University Health Science Center, Beijing 100191, China.; 6 Yunnan Baiyao Group, Kunming 650504, China.; 7 Southwest United Graduate School, Kunming 650092, China.

**Keywords:** Hepatocellular carcinoma, Lenvatinib resistance, SAHA, AKT, combination treatment

## Abstract

Hepatocellular carcinoma (HCC) is a deadly malignancy with limited treatment options. As a first-line treatment for advanced HCC, Lenvatinib has been applicated in clinic since 2018. Resistance to Lenvatinib, however, has severely restricted the clinical benefits of this drug. Therefore, it is urgent to explore the potential resistance mechanisms of Lenvatinib and identify appropriate methods to reduce resistance for the treatment of HCC. We identified SAHA, a HDAC inhibitor, to have effective anti-tumor activity against Lenvatinib-resistant HCC organoids by screening a customized drug library. Mechanism analysis revealed that SAHA upregulates PTEN expression and suppresses AKT signaling, which contributes to reversing Lenvatinib resistance in liver cancer cells. Furthermore, combinational application of Lenvatinib and HDAC inhibitor or AKT inhibitor synergistically inhibits HCC cell proliferation and induces cell apoptosis. Finally, we confirmed the synergistic effects of Lenvatinib and SAHA, or AZD5363 in primary liver cancer patient derived organoids. Collectively, these findings may enable the development of Lenvatinib combination therapies for HCC.

## Introduction

Hepatocellular carcinoma (HCC), as the most frequent primary liver cancer, is ranked as the sixth most common neoplasm and the third leading cause of cancer-related death worldwide 1. Standard treatments for early-stage HCC comprise surgical resection, transplantation, and ablation 2. However, for advanced HCC patients who are ineligible for surgical resection, systemic therapies such as Sorafenib 3, Lenvatinib 4, or Atezolizumab plus Bevacizumab 5 are commonly applicated. Lenvatinib, a multi-kinase inhibitor, was approved as a first-line treatment for patients with advanced HCC 4. Lenvatinib targets multiple kinase receptors, including VEGFR1-3, FGFR1-4, PDGFRα, RET, and KIT 6, 7. Studies have demonstrated that Lenvatinib offers comparable overall survival to Sorafenib in untreated advanced HCC, but with better progression-free survival 4. However, despite a small number of patients obtained a real and long-term benefit from this therapy, most patients do not respond or gradually develop resistance over time to Lenvatinib treatment 8. Therefore, it is crucial to acquire a comprehensive understanding of the mechanisms of Lenvatinib resistance and explore new therapeutics to overcome it.

Epigenetic regulation plays a crucial role in controlling tumor initiation and progression 9, 10. Histone deacetylases (HDACs) are essential for chromatin remodeling and regulation of gene transcription, while altered expression of HDACs actively contributes to tumor initiation and progression 11. Furthermore, HDAC inhibitors represent the pioneering success of epigenetics-based cancer therapy 12-15. The FDA has approved multiple HDACs-targeting drugs for cancer treatment. So far, four HDAC inhibitors, including SAHA, Romidepsin, Belinostat, and Panobinostat, have received FDA approval 16. For example, SAHA and Panobinostat have been approved to treat cutaneous T-cell lymphoma and multiple myeloma, respectively 17-20. Meanwhile, numerous HDAC inhibitors have been characterized to effectively suppress tumor growth and metastasis in both *in vitro* and *in vivo* models 21. Collectively, recent clinical and basic studies have indicated promising potentials of HDAC inhibitors as novel anticancer drugs 22.

The PI3K/AKT signaling pathway governs essential cellular processes, such as cell cycle regulation, survival, metabolism, motility, and angiogenesis, in physiological and pathological settings and is implicated as one of the key cancer hallmarks 23, 24. Dysregulation of the PI3K/AKT signaling pathway is frequently observed in HCC, warranting further investigation and prompting the development of novel therapeutic targets for liver cancer 25, 26. The PI3K/AKT pathway plays a critical role in mediating chemoresistance in multiple cancer types, including breast cancer, leukemia, lung cancer, ovarian cancer, melanoma, and hepatocellular carcinoma 27, 28. Additionally, activation of the PI3K/AKT pathway has been reported to promote acquired resistance to Sorafenib in HCC cells 29. Utilizing the currently available small molecule inhibitors targeting AKT presents a potential strategy to overcome tumor therapy resistance 28.

In this study, we found HDAC inhibitor SAHA could effectively suppress Lenvatinib-resistant HCC organoids' survival. Both *in vivo* and *in vitro* studies demonstrated that SAHA inhibited HCC cell proliferation, induced cell apoptosis, and enhanced the sensitivity of Lenvatinib-resistant HCC to Lenvatinib treatment via inhibiting the AKT signaling pathway. In conclusion, our findings suggest that a combination therapy of SAHA and Lenvatinib could enhance the therapeutic efficacy and impede the progression of HCC.

## Materials and Methods

### Cell lines

The human HCC cell lines, HCCLM3, SK-Hep1, HepG2, SNU-739 and the mouse HCC cell line Hepa1-6 were obtained from Key Laboratory of Cancer Prevention and Therapy, Tianjin Medical University Cancer Institute and Hospital, Tianjin Medical University (Tianjin, China). The Human HCC cell lines Hep3B, Huh7 and PLC/PRF/5 cell lines were kindly provided by Dr. Zhiqian Zhang from Peking University Cancer Hospital and Institute (Beijing, China). All Cell lines were validated by STR analysis (BIOWING, Shanghai, China). All human HCC cells and mouse liver cancer cells were cultured in Dulbecco's modified Eagle medium supplemented with 10% fatal bovine serum and 1% penicillin-streptomycin at 37 °C in a humidified atmosphere with 5% CO_2_. Mycoplasma contamination was excluded via a polymerase chain reaction-based method.

### Cell viability assays

HCC cells were plated in 96 well plates at optimized cell density. The day after cell seeding, cells were treated with compounds at indicated concentrations for 72 hours. All compounds used for cell viability assays were purchased from MedChemExpress (Shanghai, China). Cell viability was measured by sulforhodamine B assay as described previously 30. All experiments were performed in triplicate for at least twice.

### Long-term clonogenic assays

Cells were plated into 6-well plates at a density of 1-3×10^3^ cells per well. The day after cell seeding, cells were cultured in medium containing indicated drugs for 7-14 days with changing culture medium twice a week. Cells were fixed with 4% formaldehyde and stained with 0.1% crystal violet diluted in water. Crystal Violet was solubilized with 10% acetic acid, and optical results were read by an automated spectrophotometric plate reader at a wavelength of 595 nm.

### Cell apoptosis analysis

Drug treated and control cells were collected and washed by 1×PBS once. 1×10^5^ cells were stained with anti-Annexin V antibody (eBiosciences, Vienna, Austria) and propidium iodide following the manufacturer's protocol. Briefly, cells were washed with 1×binding buffer followed by staining with FITC-labeled annexin V in dark for 30 min at room temperature. Subsequently, the cells were washed with 1×binding buffer twice, and stained with propidium iodide. Stained cells were analyzed on the BD FACSCalibur (BD bioscience, NJ, USA).

### RNA sequencing and data analysis

SAHA or vehicle control treated SNU-739 cells were collected using TRIzol (Invitrogen, CA, USA) for RNA extraction and sequencing. Library preparation and RNA-seq were performed by Novogene (Beijing, China) using paired-end sequencing pipeline. Paired-end reads were generated on Illumina Hiseq 2500 platform. After quality controlling, clean data were aligned to UCSC hg19 reference by STAR and quantified using RSEM with default paraments. Differentially expressed genes were determined with the R package DESeq2. KEGG analysis were performed using ClusterProfiler package.

### AKT overexpression and siRNA transfection

The lentiviral pCDH-AKT plasmid was kindly provided by Dr. Ceshi Chen from Kunming Medical University (Kunming, China), and virus packaging was performed according to their published protocols 31. Lentiviruses infected Hep3B and Huh7 cells were selected by 1 μg/ml puromycin for further experiments. siRNAs against AKT were designed and synthesized by JTS Scientific (Beijing, China). The siRNA sequences are shown in Table S1. SNU-739 and Hepa1-6 cells were transfected with 20 nmol/L siRNA following the general transfection protocol. 36 hours after transfection, the cells were harvested for further experiments.

### Drug synergy analysis

Cells were plated in 96-well plates at 1×10^3^ cells/well, and 24 hours later, were treated with single or combined compounds as designed for 72 hours. Cell viability was measured by SRB assays. We evaluated 6 doses of Lenvatinib combined with 6 doses of SAHA (36 dose combinations) in triplicate, synergy score was analyzed and visualized with the online software Synergy Finder (version 2.0) using the Bliss model.

### Quantitative real-time PCR

Total RNA from HCC cells was isolated following TRIzol RNA extraction protocol and reverse-transcribed to cDNA using a RT-PCR kit (TianGen, Beijing, China). Quantitative PCR analysis was performed on the AriaMx Real-Time PCR System (Agilent Technologies, CA, USA) using SYBR Green PCR reagent (TianGen, Beijing, China) following the manufacturer's instructions. The sequences of primers used in our study are listed in Table S1.

### Western blotting

Whole cell lysates were prepared from cultured cells or organoids, and 30μg proteins per sample were loaded for gel separation and transferred to the PVDF membrane. The membranes were incubated with primary antibodies at 4 °C overnight. Then, the blots were incubated with the horseradish peroxidase-conjugated secondary antibody and developed by enhanced chemiluminescence. β-actin was detected as the internal control. Antibodies against PTEN, p-AKT, AKT, Cyclin D1, P21, p-BAD, BAD, Acetyl-H3, H3, and PARP were purchased from Cell Signaling Technology (MA, USA). Antibodies against β-actin, p-ERK1/2 and ERK1/2 were purchased from Santa Cruz biotechnology (CA, USA).

### ChIP assay

After SAHA or DMSO treatment, HCC cells were cross-linked with 1% formaldehyde for 10 minutes at room temperature, then lysed in SDS lysis buffer with protease inhibitors. The cell lysates were sonicated with Q125 Sonicator (QSonica, CT, USA) to obtain chromatin fragments of 300-1000 bp. Specific antibodies recognizing acetyl-histone H3K9 were employed to interact and pulldown corresponding protein-DNA fragments using protein A/G beads. The immunoprecipitated DNA was purified for further quantitative PCR analysis. The primers targeting the PTEN promoter region for qPCR assay are listed in Table S1.

### ATAC-Seq

The ATAC assay was conducted on SNU-739 cells treated with either DMSO or SAHA (5 μM) for 24 hours according to the manufacturer's protocol, utilizing the Hyperactive ATAC-Seq Library Prep Kit for Illumina (Vazyme, Nanjing, China). Subsequently, the ATAC‐seq libraries underwent paired-end sequencing on the HiSeq-PE150 platform. The ATAC-seq reads were aligned to the hg38 reference genome using Bowties, and the visualization of the PTEN locus was performed with IGV 2.17.2 software.

### In vivo mouse syngeneic graft experiments

All mice were purchased from SiPeiFu Biotechnology (Beijing, China) and housed in a 12-hour light/dark cycle with access to food and water ad libitum. For the syngeneic graft model, 5×10^5^ Hepa1-6 cells were implanted to 6-week-old C57BL/6 mice subcutaneously. Once tumors reached approximately 50 mm^3^, mice were randomized into designed groups (6 mice per group) for drug treatment as indicated. Tumor volumes were recorded every 3 days and calculated with the formula Volume (mm^3^) = L×W^2^×0.5 (L is the longest tumor axis and W is the shortest tumor axis). At the end of treatment, the mice were sacrificed and tumors were harvested for further analysis.

### Immunohistochemistry staining

The murine tumor samples were collected for Ki67 and cleaved caspase-3 detection using a standard diaminobenzidine (DAB) immunohistochemistry staining protocol as described previously. In brief, antigen-retrieved tissue slides were incubated with designed primary antibodies at 4 °C overnight followed by incubating with the biotinylated anti-mouse secondary antibody and the avidin-biotin complex (Gene Tech, Shanghai, China) sequentially. Finally, slides were incubated with DAB (Gene Tech, Shanghai, China) until suitable staining developed. Immunostaining images were taken under Axioscope 5 microscope (Carl Zeiss, Jena, Germany). Antibody against cleaved caspase-3 was purchased from Cell Signaling Technology (MA, USA). The antibody against Ki-67 was purchased from Abcam (MA, USA).

### Organoid culture and drug response assay

HCC samples used in this study were obtained during liver resection performed in Tianjin Cancer Hospital (Tianjin, China), and Henan Cancer Hospital (Zhengzhou, China). All patient samples in this study were collected with informed consent in accordance with the Declaration of Helsinki. The study was approved by hospital ethics committees (BC2020096 and 2016CT054). Detailed clinicopathological data are summarized in Table S2. Organoids were cultured according to protocols as described previously 32. Briefly, fresh liver cancer tissues were cut into small pieces, followed by incubating in DMEM/F12 containing 2mg/ml collagenase D and 0.1 mg/ml DNase I for 1 hour at 37 °C, with mechanical pipetting every 15 minutes. Digested cells (most in clusters) were filtered through 100 μm cell strainers and collected for culture. Cells were suspended in 60% matrigel diluted by complete culture media and plated in 50 μl drops into 6-well non-treated tissue culture plates and incubated for 30 min at 37 °C for gel solidification. Subsequently, 2 mL organoid culture media was added, and the medium was changed every 3 days.

For drug screening assays, collected organoids were digested by 1mg/ml dispase Ⅱ to remove Matrigel, followed by passing through a 100 μm cell strainer to remove extra-large organoids. Subsequently, organoids were resuspended in complete organoid culture medium (2×10^4^ organoids/ml) supplemented with 5% matrgel and plated into ultralow-attachment 96-well plates in triplicate. 24 h after plating, drugs were added as designed at indicated concentrations. 6 days after drug treatment, cell viability was analyzed using CellTiter-Glo 3D reagent (Promega, WI, USA) according to the manufacturer's instructions.

### Statistical analysis

Statistical analysis was performed using SPSS, version 26.0. The data are shown as mean ± SD. The significance was analyzed by Student's t-test. P values < 0.05 were considered statistically significant.

## Results

### Drug screening identifies SAHA as a potential candidate for overcoming Lenvatinib-resistance in HCC

**Figure 1:**
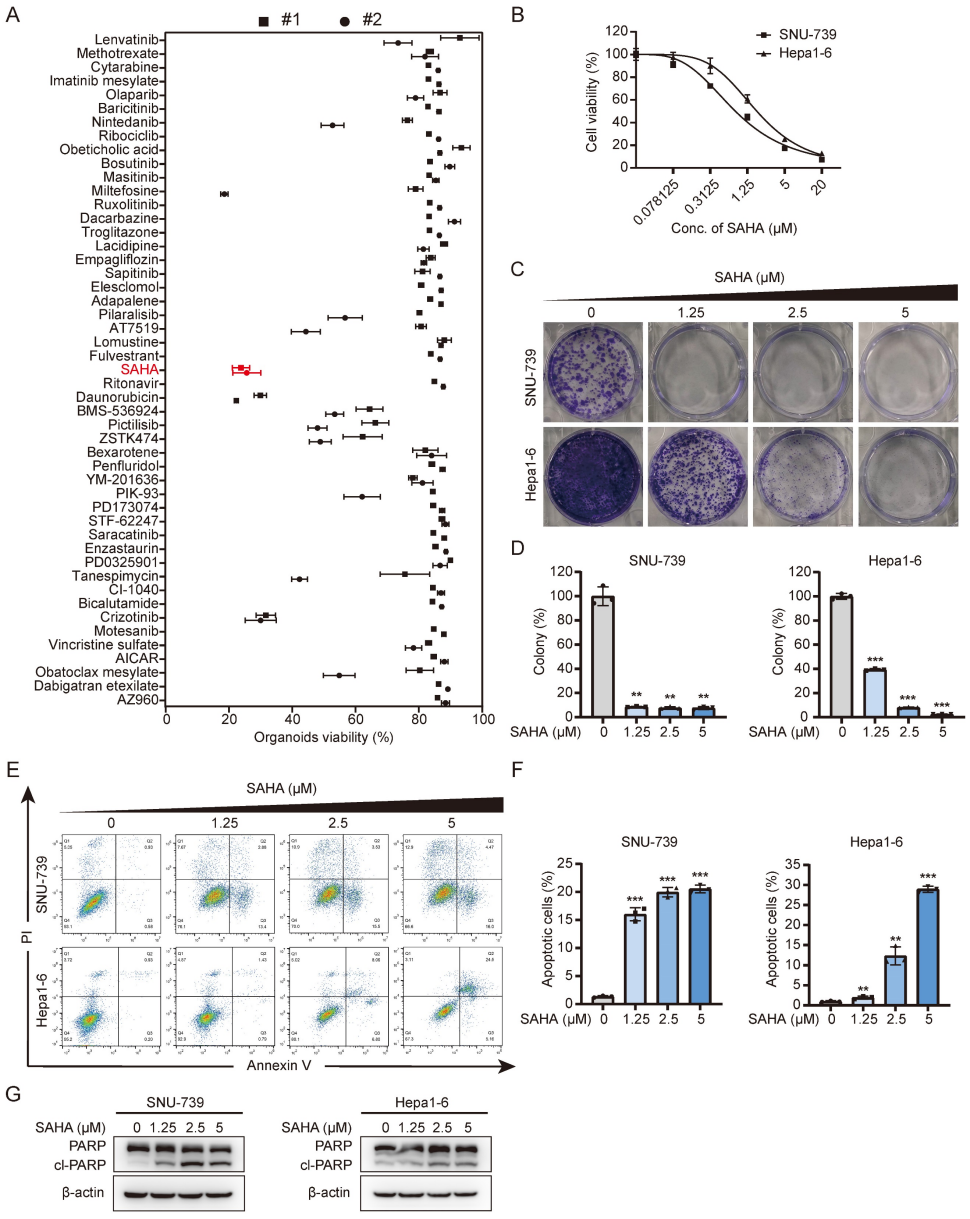
** Drug screening identifies SAHA as an effective inhibitor of HCC.** (A) Drug screening on two Lenvatinib-resistant HCC organoids. Organoids viability was assessed on Day 6 posttreatment using each compound at a concentration of 10 μM. (B) Dose-response curve of SNU-739 and Hepa1-6 cells treated with SAHA. (C) Colony formation of SNU-739 and Hepa1-6 cells in present or absent of SAHA treatment. (D) The quantification of the three independent assays shown in (C). Data are presented as mean ± SD (n = 3, *P < 0.05, **P < 0.01, ***P < 0.001, by two-sided Student's t-test). (E) Apoptosis induced by SAHA in SNU-739 and Hepa1-6 cells. Cell lines were treated as indicated for 24 hours, after which annexin V/PI staining was performed, followed by flow cytometry. (F) The quantification of the three independent assays shown in (E). Data are presented as mean ± SD (n = 3, *P < 0.05, **P < 0.01, ***P < 0.001, by two-sided Student's t-test). (G) Western blotting plots of PARP and cleaved PARP in SNU-739 and Hepa1-6 cells treated with SAHA.

In order to find drugs that can overcome Lenvatinib resistance, we selected 48 compounds, which have at least passed Phase I clinical trials with anti-cancer potentials from clinicaltrials.org, and performed drug screening using two Lenvatinib-resistant liver cancer organoids. The results showed that the HDAC inhibitor, SAHA, exhibited surprisingly effective inhibitory effects on these primary liver cancer organoids (Fig. 1A and S1A). We subsequently performed dose-response tests on 8 liver cancer cell lines with Lenvatinib and SAHA. As the results shown in figures 1 and S1, these HCC cell lines showed IC_50_s ranging from 0.601 μM to 12.55 μM to Lenvatinib treatment, while showed an average IC_50_ of 1.251±0.426 μM to SAHA, indicating these HCC cell lines are all relatively sensitive to SAHA regardless their response to Lenvatinib. We then confirmed the effects of SAHA on HCC cells using long-term clonogenic assays (Fig. 1C, 1D, S1E and S1F) and got similar results that SAHA could suppress HCC clone formation effectively at relatively low doses.

Based on the cell viability assays, we selected 2 HCC cell lines, SNU-739 and Hepa1-6, with relatively high IC_50_s to Lenvatinib treatment for further analysis (Fig. S1B and S1C). Considering both cell growth inhibition and cell death, including cell apoptosis, contribute to cell viability reduction, we also checked the effects of SAHA on HCC cell apoptosis. As shown in Figure 1E-1G, SAHA efficiently induced cell apoptosis in both SNU-739 and Hepa1-6 cells as indicated by increased Annexin V^+^ cells (Fig. 1E and 1F) and increased expression of cleaved PARP (Fig. 1G). In summary, these findings indicate that SAHA has a good inhibitory effect on Lenvatinib-resistant liver cancer cells.

### SAHA sensitizes HCC cells to Lenvatinib treatment

**Figure 2:**
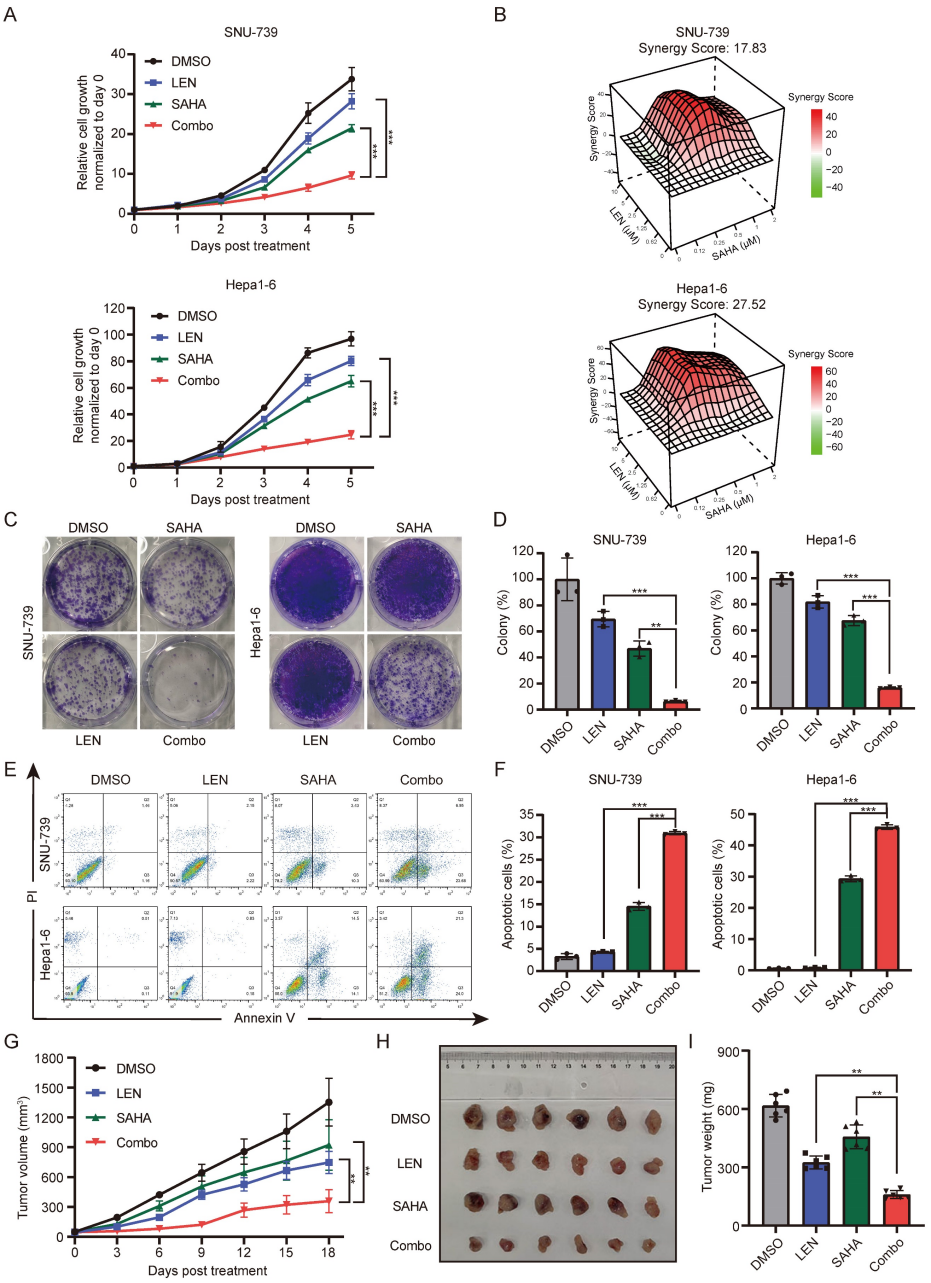
** Lenvatinib and SAHA demonstrates synergistic effects in HCC.** (A) Growth curve of SNU-739 and Hepa1-6 cells treated with either DMSO, Lenvatinib (2.5 μM), SAHA (0.5 μM) or their combination. (B) Synergy map of SNU-739 and Hepa1-6 cells treated with indicated concentrations of SAHA and Lenvatinib. (C) Colony formation of SNU-739 and Hepa1-6 cells treated with either DMSO, Lenvatinib (2.5 μM), SAHA (0.5 μM) or their combination. (D) The quantification of the three independent assays shown in (C). (E) Cell apoptosis analysis of SNU-739 and Hepa1-6 cells treated with either DMSO, Lenvatinib, SAHA or their combination. (F) The quantification of the three independent assays shown in (E). (G) Tumor growth curve of Hepa1-6 xenograft models. Mice were treated with vehicle, Lenvatinib (4 mg/kg), SAHA (40 mg/kg), or combination daily for 18 days. Growth curve was plotted by measuring the relative tumor volume every 3 days. (H-I) The excised tumors and relative tumor weight at the termination day from the experiment described in (G), respectively.

Since Lenvatinib resistant HCC cells are relatively sensitive to HDACi treatment, we wondered whether HDAC inhibition could help address the Lenvatinib resistance issues in these HCC cells. Lenvatinib, together with SAHA, were employed to treat these Lenvatinib resistant HCC cells (SNU-739 and Hepa1-6), and the synergy scores were calculated using Synergyfinder 2.0. Interestingly, application of low dose SAHA (0.5 μM) significantly sensitized these cells to 2.5 μM Lenvatinib treatment (Fig. 2A), the synergy scores of SAHA and Lenvatinib in SNU-739 and Hepa1-6 cells reached 17.83 and 27.52, respectively (Fig. 2B). In addition, except for the SK-Hep1 cells, the synergy scores of SAHA and Lenvatinib in HCCLM3, HepG2, and PLC/PRF/5 cells were all greater than 10, indicating a significant synergistic effect (Fig. S2A).

To further confirm whether SAHA can overcome Lenvatinib resistance in liver cancer cells, we employed cell growth curve analysis and long-term cloning formation assay in multiple Lenvatinib resistant cells to evaluate the combinatorial therapeutic effects of SAHA and Lenvatinib. Indeed, the combination application of SAHA and Lenvatinib significantly inhibited cell proliferation and cloning formation ability (Fig. 2C-2D and S2B-2D). Meanwhile, we identified SAHA and Lenvatinib synergistically promote apoptosis in both SNU-739 and Hepa1-6 cells (Fig. 2E-F and S2E).

Furthermore, using *in vivo* xenograft transplanting mouse model, we found combined use of SAHA and Lenvatinib significantly inhibited Hepa1-6 cell growth subcutaneously in nude mice compared with each compound alone (Fig. 2G-2I), without reducing the body weight of mice significantly (Fig. S2F). Immunohistochemical analysis of these tumor mass showed that combined drug treatment significantly suppressed Ki67 expression, and increased cleaved caspase-3 expression (Fig. S2G and S2H), indicating decreased proliferation and increased cell death of these tumors *in vivo*. Together, these results suggested that the combinatorial application of SAHA and Lenvatinib synergistically inhibits HCC tumor cell proliferation and promote cell apoptosis, both *in vitro* and *in vivo*.

### SAHA suppresses PI3K-AKT signaling by up-regulating PTEN expression

**Figure 3:**
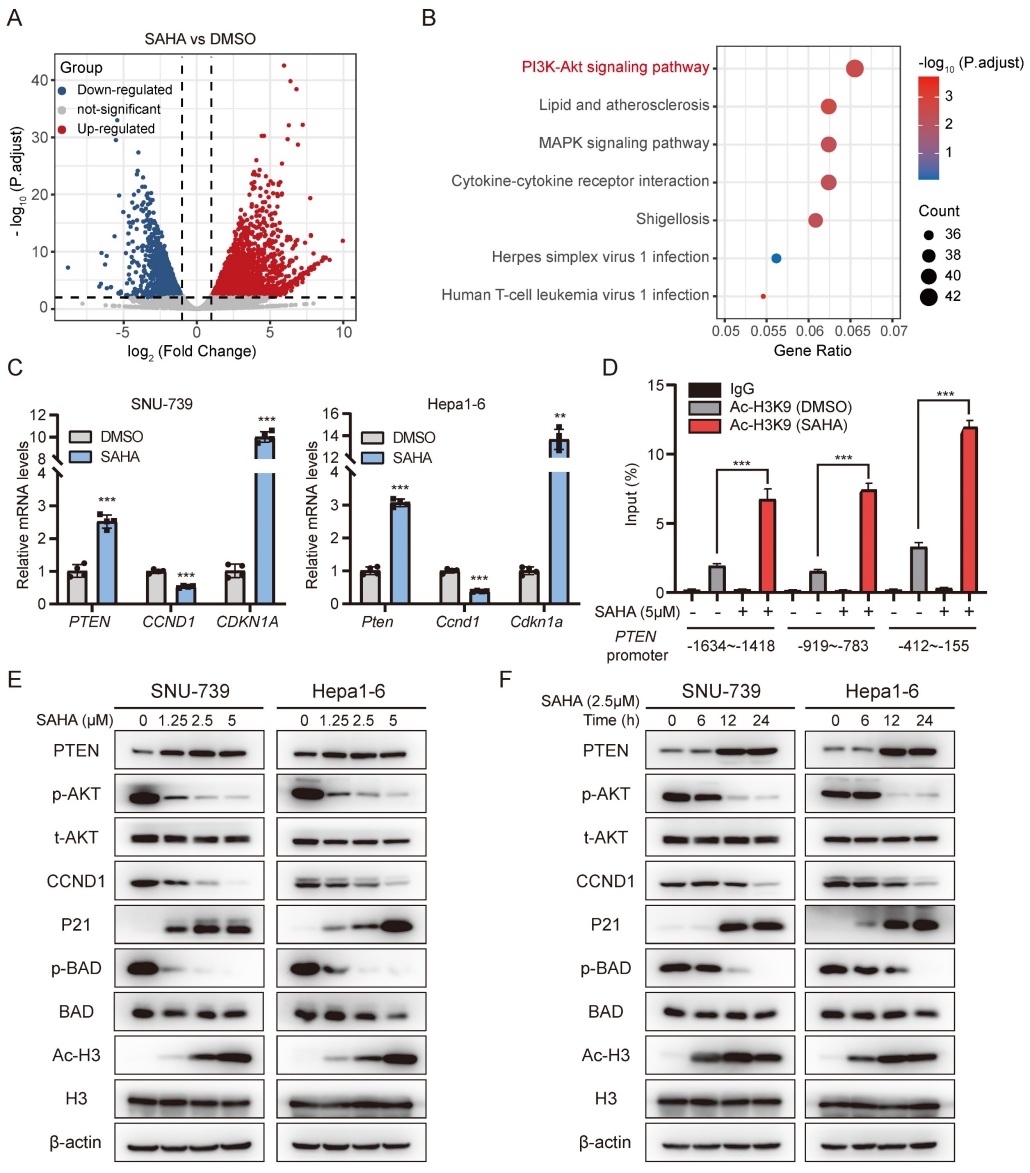
** SAHA inhibits the AKT pathway by upregulating PTEN expression.** (A) volcano plot showing differentially expressed genes in SNU-739 cells post SAHA treatment (5 μM) for 24 hours. (B) Pathway enrichment analysis of down-regulated genes in SAHA treatment cells compared to DMSO treatment cells. (C) Real-time PCR analysis of the expression of *PTEN*, *CCND1* and *CDKN1A* in SAHA treatment cells. (D) Histone acetylation in PTEN promoter. SNU-739 cells were treated with SAHA (5 μM) for 12 hours before being subjected to ChIP assay using anti-acetyl-histone H3K9 (Ac-H3K9) antibody followed by qPCR analysis using primers targeting indicated PTEN promoter region. (E-F) Western blotting analysis of the downstream of AKT signaling expression in SNU-739 and Hepa1-6 cells, which were treated with SAHA in a dose- and time-dependent manner. β-actin served as a loading control.

In order to better understand the anti-tumor mechanisms of SAHA in Lenvatinib resistant HCC, SNU-739 cells were treated with SAHA, and RNA-sequencing were performed to detect genes, pathways and regulatory networks that were dysregulated. Transcriptome analysis revealed that 1506 genes were downregulated after SAHA treatment (Fig. 3A). KEGG pathway enrichment analysis identified the PI3K-AKT signaling pathway as the most downregulated pathway in SAHA-treated SNU-739 cells (Fig. 3B). Detailed analysis of PI3K-AKT signaling revealed genes, including *PTEN* and *CDKN1A* are upregulated, while *CCND1* is downregulated in this pathway (Fig. S3A). We validated these genes' expression using RT-PCR in SAHA-treated SNU-739 and Hepa1-6 cells, and confirmed these genes had the same changing trends in both cell lines (fig. 3C).

It's well-documented that HDAC mainly works through removing acetyl groups from DNA-binding histone proteins, which is generally associated to a decrease in chromatin accessibility for transcription factors and repressive effects on gene expression. We then checked whether SAHA promotes target genes' expression, such as *PTEN*, by opening chromatin structure. We performed chromatin immunoprecipitation (ChIP) assay using acetyl-histone H3K9 antibody in SAHA-treated SNU-739 cells, followed by qPCR analysis. As expected, SAHA treatment significantly enriched the presence of the *PTEN* gene promoter region in the precipitant pulled down by Ac-H3K9, as probed by three independent pairs of primers specifically targeting *PTEN* gene promoter, indicating SAHA treatment increased histone acetylation in *PTEN* promoter region (Fig. 3D). To further confirm the impact of SAHA on *PTEN* transcription regulation, we utilized ATAC-Seq to analyze the chromatin accessibility of the PTEN promoter region in SNU-739 cells after SAHA treatment. Regions of accessible chromatin surrounding PTEN were increased by SAHA treatment, especially in the promoter region (Fig. S3B). SAHA is a pan-inhibitor for multiple HDACs, especially subtype I (HDAC1, 2, 3, and 8), IIa (HDAC4, 5, 7, and 9), IIb (HDAC6, and 10) and IV (HDAC11) 33. To investigate which HDAC is responsible for regulating PTEN expression, we depleted HDAC1-11 in SNU-739 cells using siRNAs targeting each HDAC respectively. As the results shown in supplementary figures 3C-E, PTEN expression is significantly increased upon silencing either HDAC1, HDAC3, HDAC4, or HDAC7, while no obvious effects were observed when other HDACs were depleted, indicating SAHA may suppress PTEN expression by targeting one or multiple members of HDAC1, HDAC3, HDAC4, and HDAC7. Moreover, immunoblotting results also showed SAHA treatment led to attenuated ATK phosphorylation, Cyclin D1 expression, and increased P21 expression in a dose- and time-dependent manner (Fig. 3E and 3F). These data suggest SAHA treatment inhibits the AKT signaling pathway in HCC cells.

### Activated PI3K-AKT pathway contributes to Lenvatinib resistance of HCC

**Figure 4:**
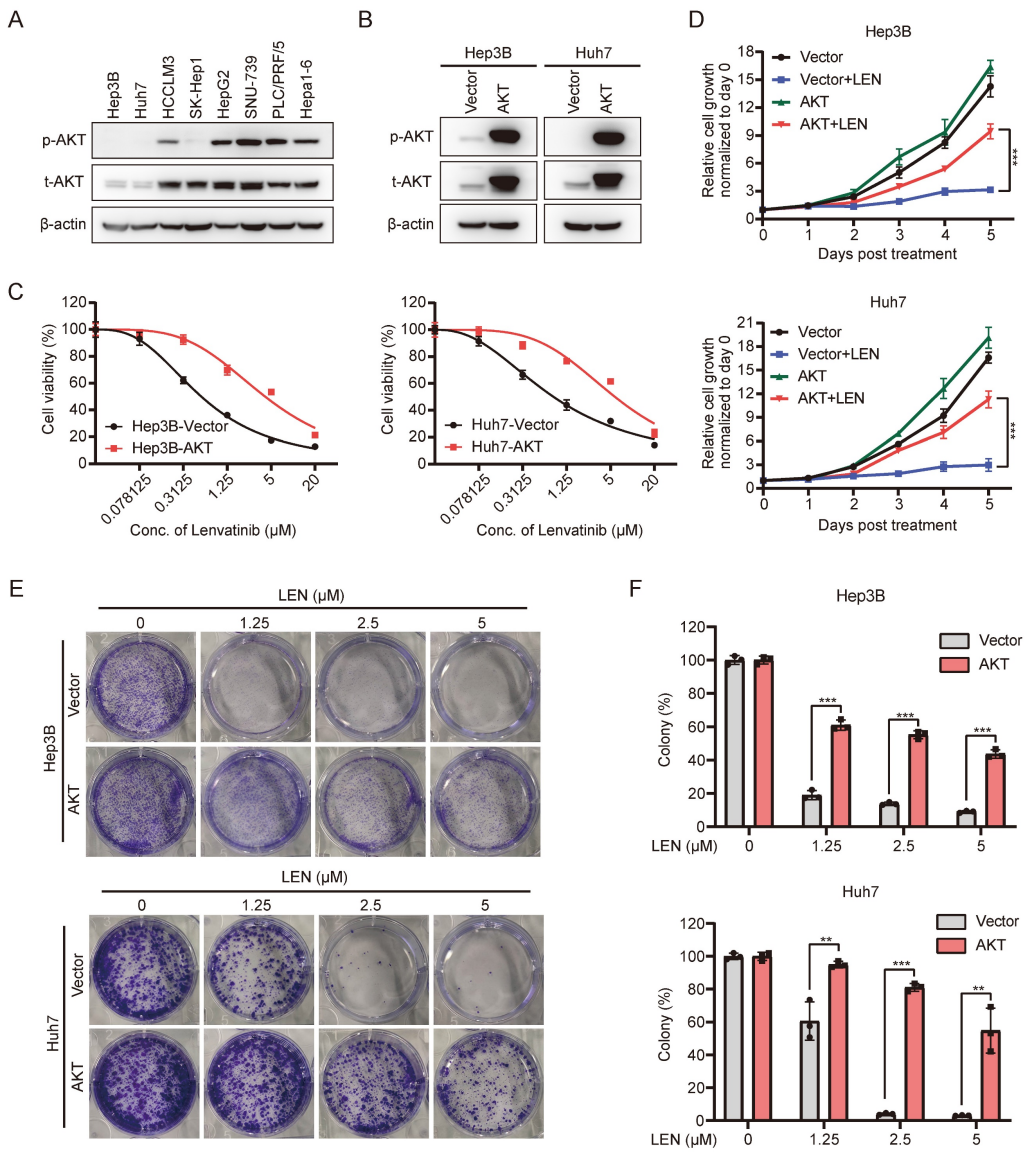
** Activation of the AKT pathway leads to resistance to Lenvatinib in HCC.** (A) Western blotting analysis of the expression of total AKT and p-AKT in a panel of liver cancer cell lines. (B) Western blotting analysis of AKT and p-AKT expression in Hep3B and Huh7 cells with transduction of pCDH-AKT lentiviral particles. (C) Dose-response curves of Hep3B and Huh7 cells with vector or AKT overexpression treated with Lenvatinib. (D) Growth curve of control or AKT overexpressed Hep3B and Huh7 cells treated with Lenvatinib (2.5 μM). (E) Colony formation assay in Hep3B and Huh7 cells treated with Lenvatinib after overexpressed AKT or vector. (F) The quantification of the three independent assays shown in (E).

Aberrant activation of PI3K-AKT pathway is associated with tumorigenesis, cancer progression, and drug resistance in various types of malignancies, and is considered as one of the most effective targets for cancer therapy 28. In order to investigate whether PI3K-AKT signaling contributes to Lenvatinib resistance of HCC, we first conducted transcriptome analysis using publicly available data (GSE211850). Compared to Huh7 parental cells, we observed enrichment of the PI3K/AKT signaling pathway in Lenvatinib-resistant Huh7 cells (Fig. S4A and S4B). Subsequently, we examined the expression of AKT in HCC cell lines, and found that AKT and p-AKT expression were relatively higher in Lenvatinib-resistant cell lines compared to sensitive ones (Fig. 4A, S1B and S1C). We further ectopically overexpressed the AKT in Lenvatinib-sensitive Hep3B and Huh7 cell lines (Fig. 4B and S4C), and found AKT overexpressed cells are more resistant to Lenvatinib treatment (Fig. 4C-4F).

**Figure 5:**
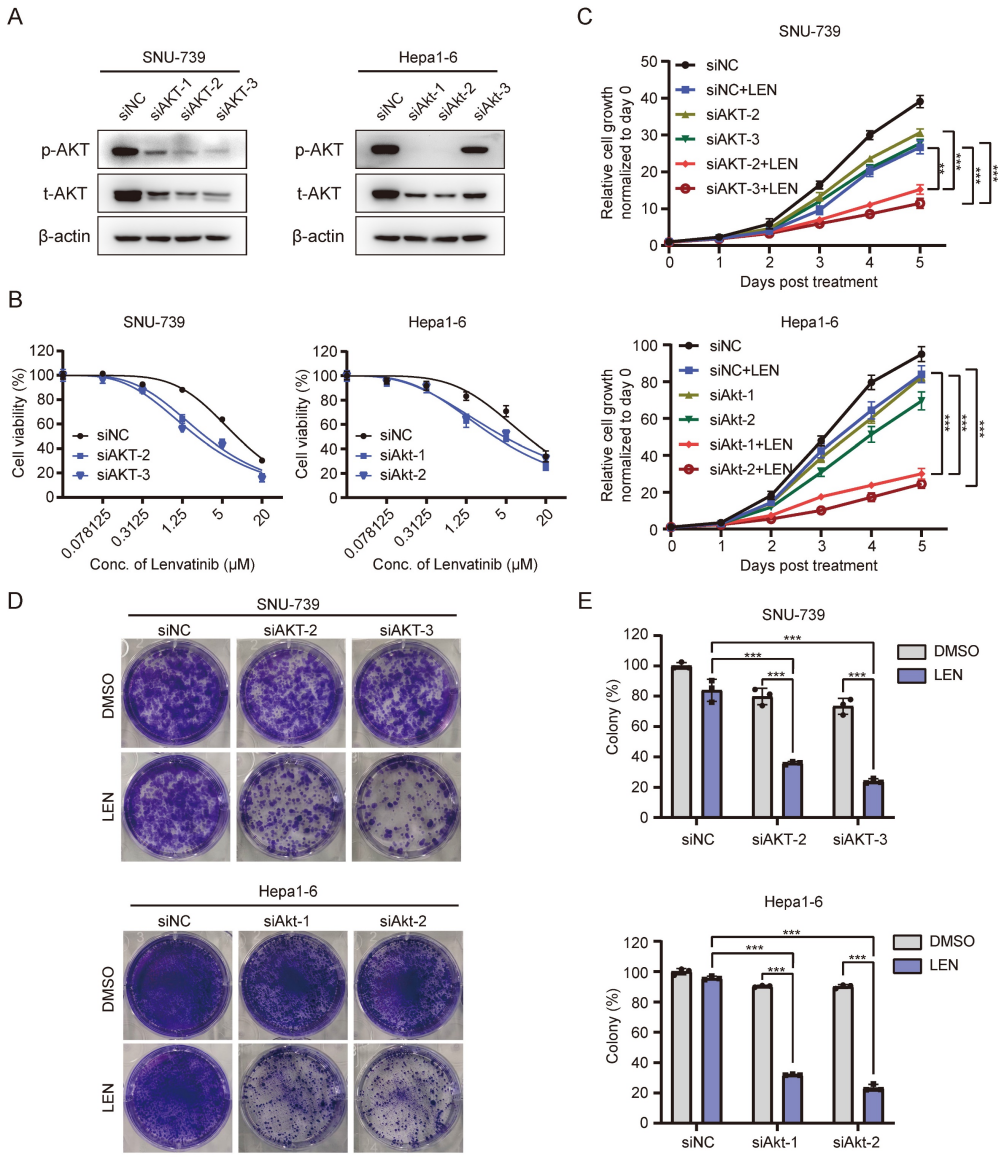
** Inhibition of the AKT pathway reverse Lenvatinib resistance in HCC.** (A) Western blotting analysis of AKT and p-AKT expression in SNU-739 and Hepa1-6 cells transfected with siNC and siAKT. (B) Dose-response curves of SNU-739 and Hepa1-6 cells treated with Lenvatinib after transfection with either siNC or siAKT. (C) The growth curves show the fold change of SNU-739 and Hepa1-6 with siNC or siAKT treated with Lenvatinib (2.5 μM). (D) Colony formation assay in SNU-739 and Hepa1-6 cells treated with Lenvatinib (2.5 μM) after knockdown AKT. (E) The quantification of the three independent assays shown in (D).

Since AKT activation contributes to Lenvatinib-resistance in HCC cells, we wondered whether blocking such signaling might facilitate overcoming HCC cell resistance to Lenvatinib. Interestingly, AKT depletion (Fig. 5A and S4D) significantly enhanced SNU-739 and Hepa1-6 cells' sensitivity to Lenvatinib treatment (Fig. 5B). Cell proliferation and long-term colony formation assays yielded consistent results (Fig. 5C-E). As aforementioned, AKT overexpression conferred resistance to Lenvatinib in Hep3B and Huh7 cells, however, treatment with SAHA markedly decreased AKT phosphorylation level (Fig. S4E and S4F) and partially restored sensitivity to Lenvatinib (Fig. S5A-5C), which further confirmed AKT inhibition sensitizes HCC cells to Lenvatinib. These data suggest AKT as an important mediator to Lenvatinib resistance.

### AKT inhibitor, AZD5363, sensitizes HCC cells to Lenvatinib

**Figure 6:**
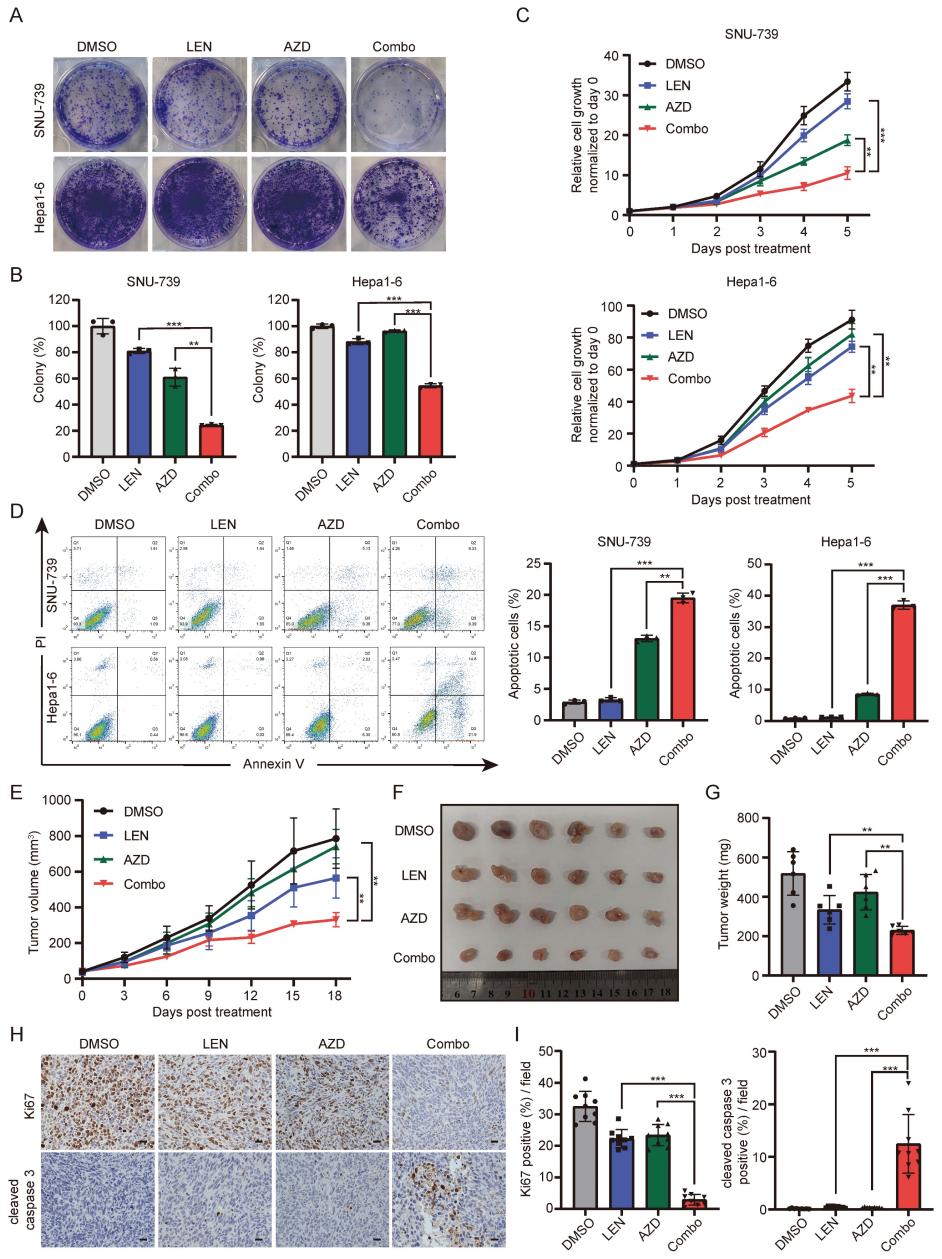
** The AKT inhibitor AZD5363 synergizes with Lenvatinib in HCC.** (A) Colony formation of SNU-739 and Hepa1-6 cells treated with either DMSO, Lenvatinib (2.5 μM), AZD5363 (1.25 μM) or their combination. (B) The quantification of the three independent assays shown in (A). (C) Growth curve of SNU-739 and Hepa1-6 treated with either DMSO, Lenvatinib (2.5 μM), AZD5363 (1.25 μM) or combination. (D) Cell apoptosis analysis of SNU-739 and Hepa1-6 cells treated with either DMSO, Lenvatinib, AZD or combination. (E) Tumor growth curve of Hepa1-6 xenograft models. Mice were treated with vehicle, Lenvatinib (4 mg/kg), AZD5363 (100 mg/kg), or combination daily for 18 days. Growth curve was plotted by measuring the relative tumor volume every 3 days. (F-G) The excised tumors and relative tumor weight at the termination day from the experiment described in E, respectively. (H) Representative images of Ki-67 and cleaved caspase-3 staining in Hepa1-6 xenograft models. Scale bars, 25 μm. (I) Quantification of Ki67 or cleaved caspase-3 positive rate of tumor from mice in each group.

Based on the above results that ATK depletion attenuated HCC cell resistance to Lenvatinib, we further employed a well-validated AKT inhibitor, AZD5363, to further confirm whether AKT inhibition contributes to overcome Lenvatinib resistance. It did show that combination of AZD5363 and Lenvatinib was more effective in suppressing cell survival than each single-drug, indicating a synergistic effect between AZD5363 and Lenvatinib (Fig. 6A-6C). Additionally, we performed cell apoptosis assays, which revealed that the combined treatment significantly promoted apoptosis in SNU-739 and Hepa1-6 cells, accompanied by an increased expression of cleaved PARP (Fig. 6D and S6A). Using syngeneic graft mouse model, we evaluated the combination effects of the two drugs *in vivo*. Compared to the groups treated with AZD5363 or Lenvatinib alone, the combined application of both drugs resulted in a significant reduction in tumor volume (Fig. 6E-6G), without reducing mouse weight compared to vehicle control (Fig. S6B). Immunohistochemistry results showed a significantly decreased Ki67 and increased cleaved caspase 3 positive rates in the combination group (Fig. 6H and 6I). Collectively, these data suggest that the AKT inhibitor AZD5363, in combination with Lenvatinib, exhibits synergistic effects and can reverse Lenvatinib resistance in liver cancer cells.

**Figure 7:**
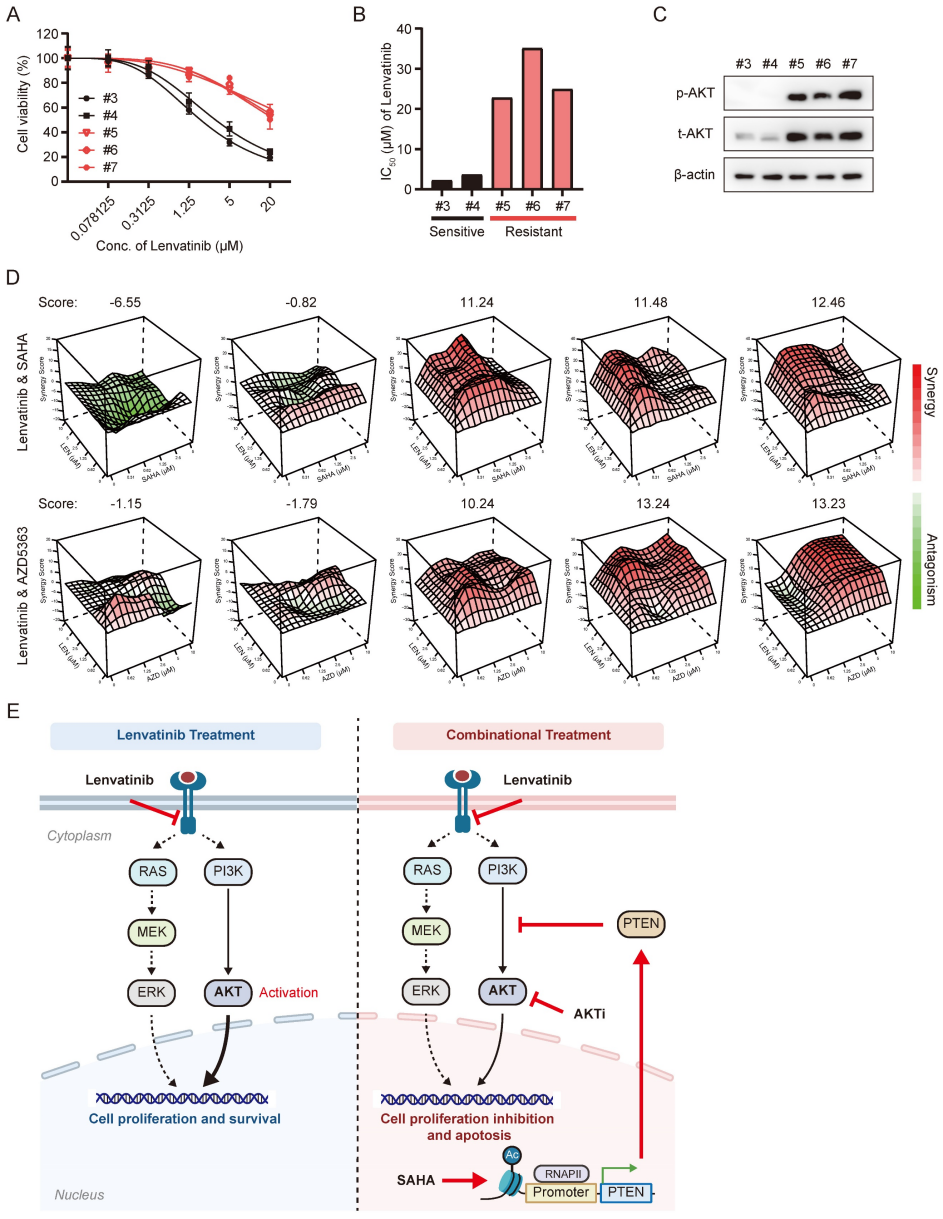
** The synergistic effects of Lenvatinib with SAHA or AZD5363 were validated in HCC organoid models.** (A) Dose-response curve of HCC organoids treated with Lenvatinib. (B) Average IC_50_ values generated from dose-response curves from (A). (C) Western blotting analysis of the expression of total AKT and p-AKT in HCC organoids. (D) Synergy map of HCC organoids treated with the combination of Lenvatinib and SAHA or AZD5363. (E) Schematic models of the mechanism underlying SAHA or AKT inhibitor reverses Lenvatinib resistance in HCC. Lenvatinib mainly reduced the MAPK pathway signaling while activation of the AKT pathway allows cells to continue to proliferate and survive independent of MAPK signaling, blocking AKT signaling pathway by either HDACi or AKT inhibitor resensitized Lenvatinib resistant HCC to Lenvatinib treatment.

In order to explore the clinical relevance of combinatorial application of SAHA and Lenvatinib, we tested the combination effects of these two drugs in liver cancer organoids. We performed dose-response tests on 5 primary liver cancer organoids with Lenvatinib (Figure 7A-B), which showed 2 of them (with IC_50_s less than 5 μM) were relatively sensitive to Lenvatinib treatment, while 3 of them were resistant to Lenvatinib (with IC_50_s greater than 20 μM). Interestingly, the p-AKT level were much higher in Lenvatinib-resistant organoids compared to sensitive ones (Figure 7C). Moreover, both the combinatorial application of SAHA/Lenvatinib, and AZD5363/Lenvatinib, exhibited extraordinarily better inhibitory effects than Lenvatinib alone in Lenvatinib-resistant tumor organoids, but not in Lenvatinib-sensitive ones (Fig. 7D and S7). These results support our hypothesis that Lenvatinib exerts anticancer effects partially by inhibiting MEK/ERK signaling pathway via targeting receptor tyrosine kinases, while AKT signaling activation attenuates HCC cells' sensitivity to Lenvatinib. Thus, targeting AKT by either HDACi or AKT inhibitor showed significant efficacy in Lenvatinib-resistant HCC cells and organoid, which may benefit HCC patients with AKT activation (Fig. 7E).

## Discussion

HCC is considered as one of the most challenging malignancies owing to its high incidence and mortality rates 1. Lenvatinib has been approved as a first-line treatment in 2018 for unresectable or advanced HCC due to its non-inferior cure rate and improvement in progression-free survival compared to sorafenib 4. Nevertheless, a significant proportion of HCC patients either exhibit no response to Lenvatinib or develop resistance during drug treatment, thereby diminishing its efficacy 34. Hence, a comprehensive understanding of the molecular mechanisms underlying Lenvatinib resistance, the exploration of effective therapeutic strategies, are of utmost importance to improve treatment response for HCC patients.

Lenvatinib is a well-defined tyrosine kinase inhibitor for multiple targets, including receptors VEGFR1-3, FGFR1-4, PDGFR, KIT, and RET 6. These targets are well-demonstrated to promote cell growth, proliferation, and angiogenesis through activating various signaling, including MAPK and PI3K/AKT pathways, in HCC 35. Numerous studies have determined various factors associated with Lenvatinib resistance, such as Jin et al. found that inhibition of receptor tyrosine kinases by Lenvatinib leads to feedback activation of EGFR-PAK2-ERK5 pathways, which reduced the effectiveness of Lenvatinib in HCC 36. Besides, other mechanisms involved in cell metabolism 37, epigenetics 38-40, autophagy 41, 42, EMT 43, noncoding RNAs 44-47, and the tumor microenvironment 48, have also been suggested to be related to HCC Lenvatinib resistance.

Several HDAC members, including HDAC1, HDAC2, HDAC4, HDAC7, etc., have been reported to correlated with HCC progression and patient survival 49, and HDAC2 expression is upregulated in Lenvatinib-resistant cells 50, suggesting a potential role of HDACs in Lenvatinib resistance. Accordingly, through a pharmacological screen of potential compounds that might conquer Lenvatinib-resistance in liver cancer, we identified SAHA as a candidate using Lenvatinib-resistant organoids and confirmed this result in more cell lines and organoids. HDAC inhibitors, like other epigenetic-based therapeutic strategies, induce extensive transcriptional alterations, and subsequent growth arrest, differentiation inhibition and cell death in cancer cells, thus exhibit anti-tumor efficacy in a variety of cancer types 11. Although HDACis have shown very positive pre-clinical results and four compounds have received clinical approval, only limited success was achieved when used as mono-therapeutic agents against solid tumors in clinical trials compared to the hematological malignancies 51. Meanwhile, side effects and toxicities from HDACis also hinder their progress in the clinic 52, 53. Thus, administering HDACis in low doses and, meanwhile, in combination with appropriate compounds might mitigate the drug toxicity and optimize curative effects simultaneously. Therefore, current clinical trials frequently combine HDAC inhibitors with chemotherapy or other targeted therapies to enhance clinical efficacy. We also noticed in our study that application of low dose SAHA did not obviously suppress HCC cell viability in mouse and organoid models (Figs. 2H and S7), while combinatorial use of low dose SAHA and Lenvatinib severely reduced cell viabilities.

We further revealed PTEN/PI3K/AKT pathway as the major signaling that was down-regulated by HDACi in Lenvatinib-resistant cells, suggesting activation of such pathway contributes to Lenvatinib resistance of HCC. The PI3K/AKT signaling pathway is considered to be one of the most crucial pathways involved in cancer initiation and progression 23, and several studies have underlined a potential role of PI3K/AKT activation in promoting drug resistance of HCC 29, 54. Indeed, we found ectopic overexpression of a constitutive active form of AKT in Lenvatinib sensitive HCC cells promotes cell resistance to Lenvatinib treatment (Fig. 4), while depletion of AKT in Lenvatinib resistant HCC cells restored sensitivity to Lenvatinib treatment (Fig. 5). Even so, we also noticed HDACi achieved better tumor-suppression efficacy than AKT inhibition in both *in vitro* and *in vivo*, in both single and combinatorial treatment (Fig. 2 and Fig. 6), suggesting HDACis might also function through inhibiting signaling pathways other than PI3K/AKT pathway, such as MAPK signaling pathway (Fig. 3B).

Taken together, we identified HDACi SAHA as a potential resolution for overcoming HCC Lenvatinib resistance via an HCC organoid-based drug screening in this study. Further mechanism research revealed PTEN/PI3K/AKT pathway as the major signaling in mediating Lenvatinib-resistant. Our data indicated that a subset of HCC cell lines or organoids displaying high levels of AKT phosphorylation exhibits resistance to Lenvatinib. More importantly, either HDACi or AKTi could sensitize these HCC cells to Lenvatinib, the combination of Lenvatinib with SAHA or AZD5363 targets the AKT signaling pathway could overcome Lenvatinib resistance. Our results offer substantial experimental evidences and comprehensive mechanistic investigation to support a novel combination therapy strategy for the treatment of advanced HCC.

## Supplementary Material

Supplementary figures and tables.
