# Supplementary material for: HDAC Inhibition Sensitize Hepatocellular Carcinoma to Lenvatinib via Suppressing AKT Activation
Source: Int J Biol Sci. 2024 May 19;20(8):3046–60. doi: 10.7150/ijbs.93375 (PMC11186361; doi:10.7150/ijbs.93375)
Supplement: Supplementary file 1 — Supplementary figures and tables. [file ijbsv20p3046s1.pdf]

Supplementary Figure Legends

Figure S1. SAHA demonstrates inhibitory effects on HCC cell lines and organoids. (A) Representative bright-field images of two HCC organoids on Day 6 posttreatment with 10 μM Lenvatinib or SAHA. (B) Dose-response curve of HCC cell lines treated with Lenvatinib at indicated dosages. (C) Average IC_{50} values generated from dose-response curves from (B). (D) Dose-response curve of HCC cell lines treated with SAHA. (E-F) Colony formation of HCC cell lines treated with SAHA at indicated dosages.

Figure S2. The combination of Lenvatinib and SAHA exhibits synergistic effects in HCC. (A) Synergy map of Lenvatinib-resistant HCC cell lines treated with SAHA and Lenvatinib at indicated concentrations. (B) Growth curve of Lenvatinib-resistant HCC cells treated with either DMSO, Lenvatinib (2.5 μM), SAHA (0.5 μM) or in combination. (C-D) Colony formation of Lenvatinib-resistant cells treated with either DMSO, Lenvatinib (2.5 μM), SAHA (0.5 μM) or in combination. (E) Western blotting plots of PARP and cleaved PARP in SNU-739 and Hepa1-6 cells treated with Lenvatinib, SAHA or in combination. (F) The body weight of mice in the Hepa1-6 xenograft model. (G-H) Ki-67 and cleaved caspase-3 staining in Hepa1-6 xenografts. Scale bars, 25 μm.

Figure S3. SAHA inhibits the AKT pathway by enhancing PTEN expression. (A) Heatmap of PI3K/AKT pathway related genes. (B) Gene tracks depicting ATAC-Seq signal at *PTEN* locus in SNU-739 cells treated with DMSO or SAHA (5 μM). (C) RT-qPCR analysis of *HDACs* expression in SNU-739 cells. (D) RT-qPCR analysis of *PTEN* expression in HDACs silenced SNU-739 cells. (E) Western blotting analysis of PTEN expression in SNU-739 transfected with control siRNA or siHDACs.

Figure S4. AKT activation is correlated with the development of resistance to Lenvatinib. (A) Volcano plot showing differentially expressed genes in Huh7 Lenvatinib-resistant cells compared to Huh7 parental cells. (B) Pathway enrichment analysis of up-regulated genes in Huh7 Lenvatinib-resistant cells compared to Huh7

parental cells. (C) RT-qPCR analysis of *AKT* expression in AKT overexpressed Hep3B and Huh7 cells. (D) RT-qPCR analysis of *AKT* expression in AKT silencing SNU-739 and Hepa1-6 cells. (E) Western blotting analysis of the downstream of AKT signaling and ERK expression in SNU-739 and Hepa1-6 cells, which were treated with Lenvatinib, SAHA or their combination. (F) Western blotting analysis of the downstream of AKT signaling and ERK expression in Hep3B and Huh7 cells with AKT overexpression, which were treated with Lenvatinib, SAHA or their combination.

Figure S5. SAHA overcomes Lenvatinib resistance by targeting the AKT signaling pathway. (A) Growth curve of control or AKT overexpressed Hep3B and Huh7 cells treated with either DMSO, Lenvatinib (2.5 μM), SAHA (0.5 μM) or in combination. (B-C) Colony formation control or AKT overexpressed Hep3B and Huh7 cells treated with either DMSO, Lenvatinib (2.5 μM), SAHA (0.5 μM) or in combination.

Figure S6. AZD5363 synergistically enhances the inhibitory effects of Lenvatinib in HCC. (A) Western blotting analysis of the downstream targets of AKT signaling and ERK expression in SNU-739 and Hepa1-6, which were treated with Lenvatinib, AZD5363 or in combination. (B) The body weight of mice in the Hepa1-6 xenograft model after treatment with indicated drugs.

Figure S7. SAHA or AZD5363 sensitizes Lenvatinib resistant HCC organoids to Lenvatinib treatment. Brightfield images of organoids that were treated for 6 days with Lenvatinib (5 μM), SAHA (1.25 μM), AZD5363 (2.5 μM) and in combinations.

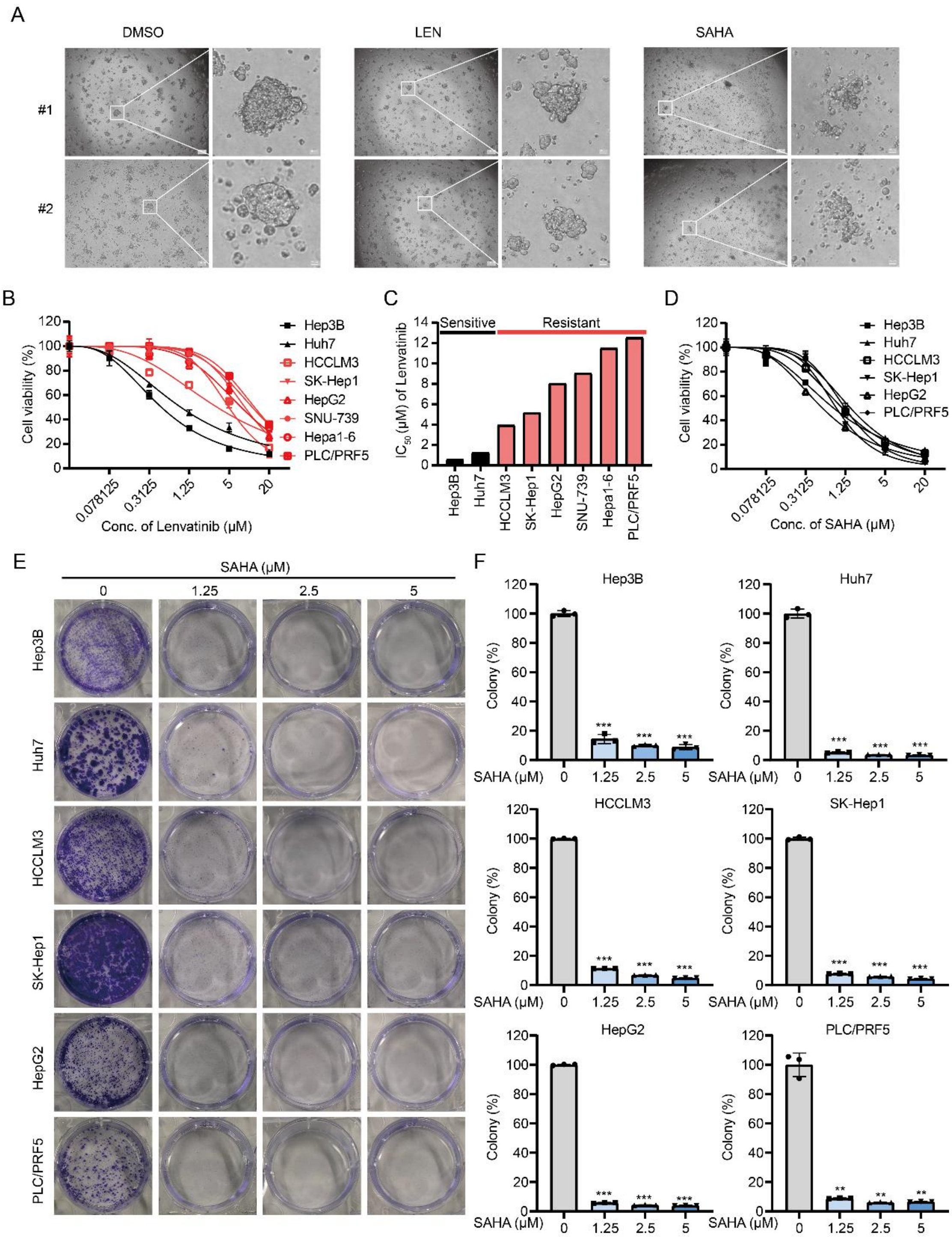
A
DMSO
LEN
SAHA
#1
#2
B
Cell viability (%)
Conc. of Lenvatinib (μM)
Hep3B
Huh7
HCCLM3
SK-Hep1
HepG2
SNU-739
Hepa1-6
PLC/PRF5
C
Sensitive
Resistant
IC50 (μM) of Lenvatinib
D
Cell viability (%)
Conc. of SAHA (μM)
E
SAHA (μM)
F
Colony (%)
SAHA (μM)

Figure S1

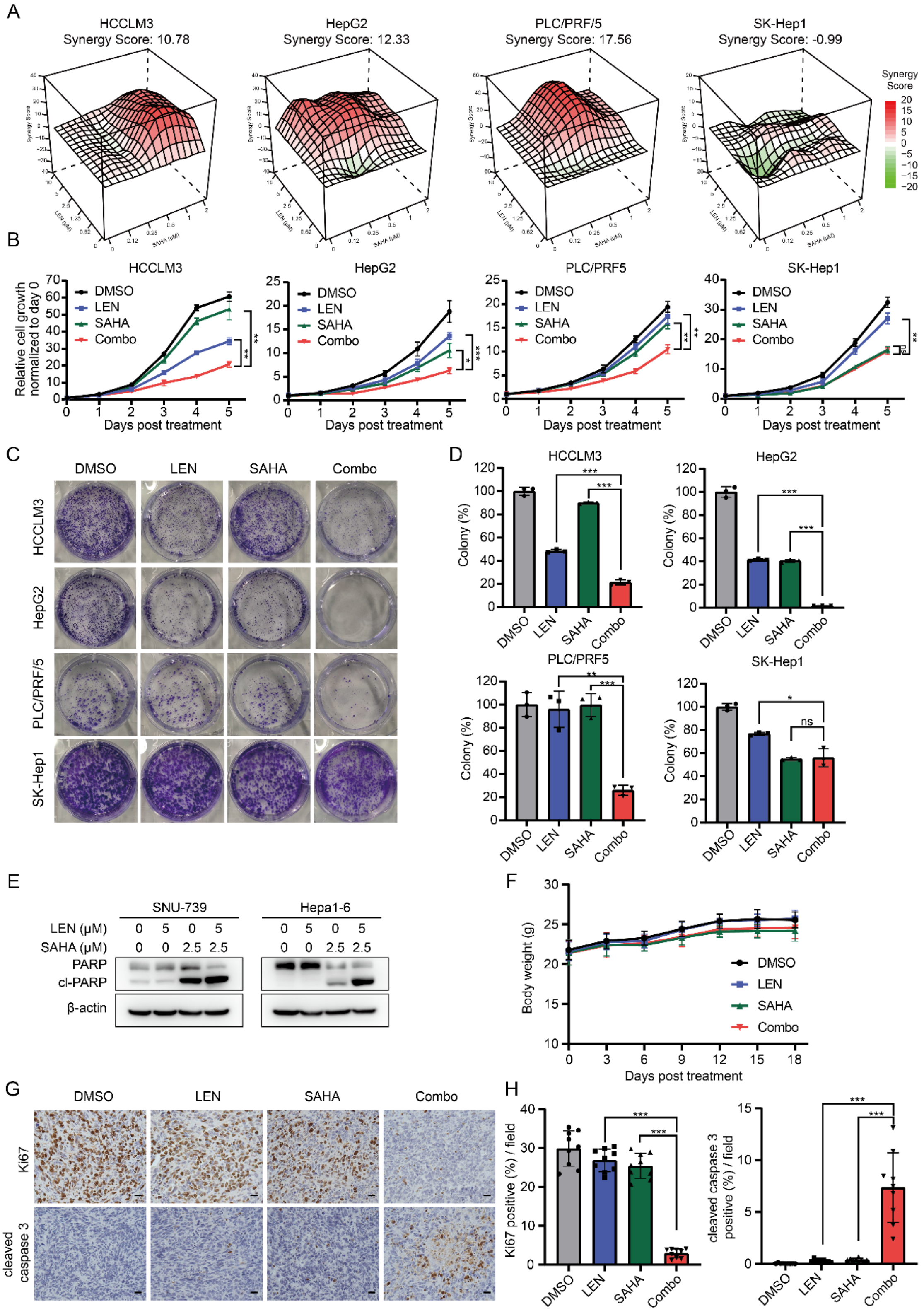
A
HCCLM3
Synergy Score: 10.78
HepG2
Synergy Score: 12.33
PLC/PRF/5
Synergy Score: 17.56
SK-Hep1
Synergy Score: -0.99
Synergy Score
B
Relative cell growth normalized to day 0
Days post treatment
DMSO
LEN
SAHA
Combo
C
D
Colony (%)
E
SNU-739
Hepa1-6
LEN (μM)
SAHA (μM)
PARP
cl-PARP
β-actin
F
Body weight (g)
G
Ki67
cleaved caspase 3
H
Ki67 positive (%) / field
cleaved caspase 3 positive (%) / field

Figure S2

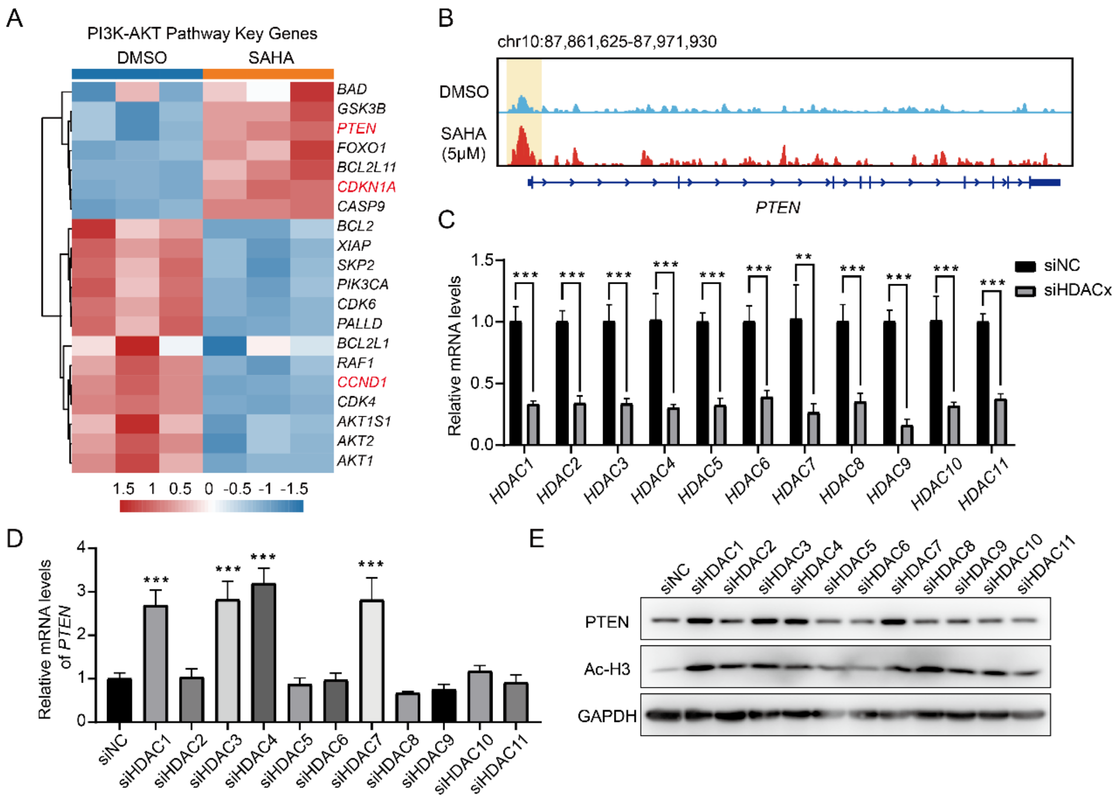
A
PI3K-AKT Pathway Key Genes
DMSO
SAHA
BAD
GSK3B
PTEN
FOXO1
BCL2L11
CDKN1A
CASP9
BCL2
XIAP
SKP2
PIK3CA
CDK6
PALLD
BCL2L1
RAF1
CCND1
CDK4
AKT1S1
AKT2
AKT1
1.5 1 0.5 0 -0.5 -1 -1.5
B
chr10:87,861,625-87,971,930
DMSO
SAHA (5μM)
PTEN
C
Relative mRNA levels
siNC
siHDACx
HDAC1
HDAC2
HDAC3
HDAC4
HDAC5
HDAC6
HDAC7
HDAC8
HDAC9
HDAC10
HDAC11
D
Relative mRNA levels of PTEN
siNC
siHDAC1
siHDAC2
siHDAC3
siHDAC4
siHDAC5
siHDAC6
siHDAC7
siHDAC8
siHDAC9
siHDAC10
siHDAC11
E
PTEN
Ac-H3
GAPDH

Figure S3

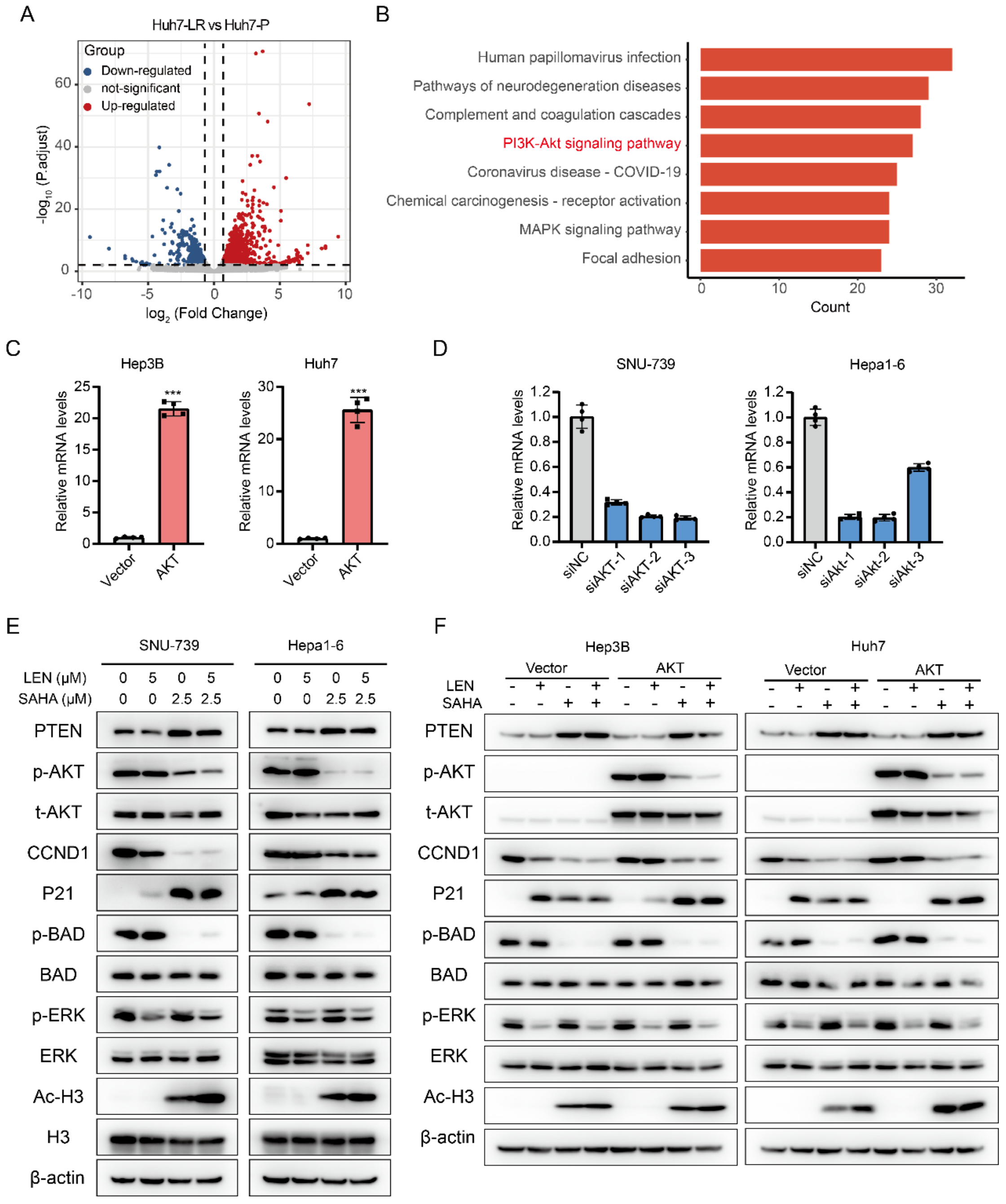
A
Huh7-LR vs Huh7-P
Group
Down-regulated
not-significant
Up-regulated
-log10 (P.adjust)
log2 (Fold Change)
B
Human papillomavirus infection
Pathways of neurodegeneration diseases
Complement and coagulation cascades
PI3K-Akt signaling pathway
Coronavirus disease - COVID-19
Chemical carcinogenesis - receptor activation
MAPK signaling pathway
Focal adhesion
Count
C
Hep3B
Huh7
Relative mRNA levels
Vector
AKT
D
SNU-739
Hepa1-6
Relative mRNA levels
siNC
siAKT-1
siAKT-2
siAKT-3
siAkt-1
siAkt-2
siAkt-3
E
SNU-739
Hepa1-6
LEN (μM)
SAHA (μM)
PTEN
p-AKT
t-AKT
CCND1
P21
p-BAD
BAD
p-ERK
ERK
Ac-H3
H3
β-actin
F
Hep3B
Huh7
Vector
AKT
LEN
SAHA

Figure S4

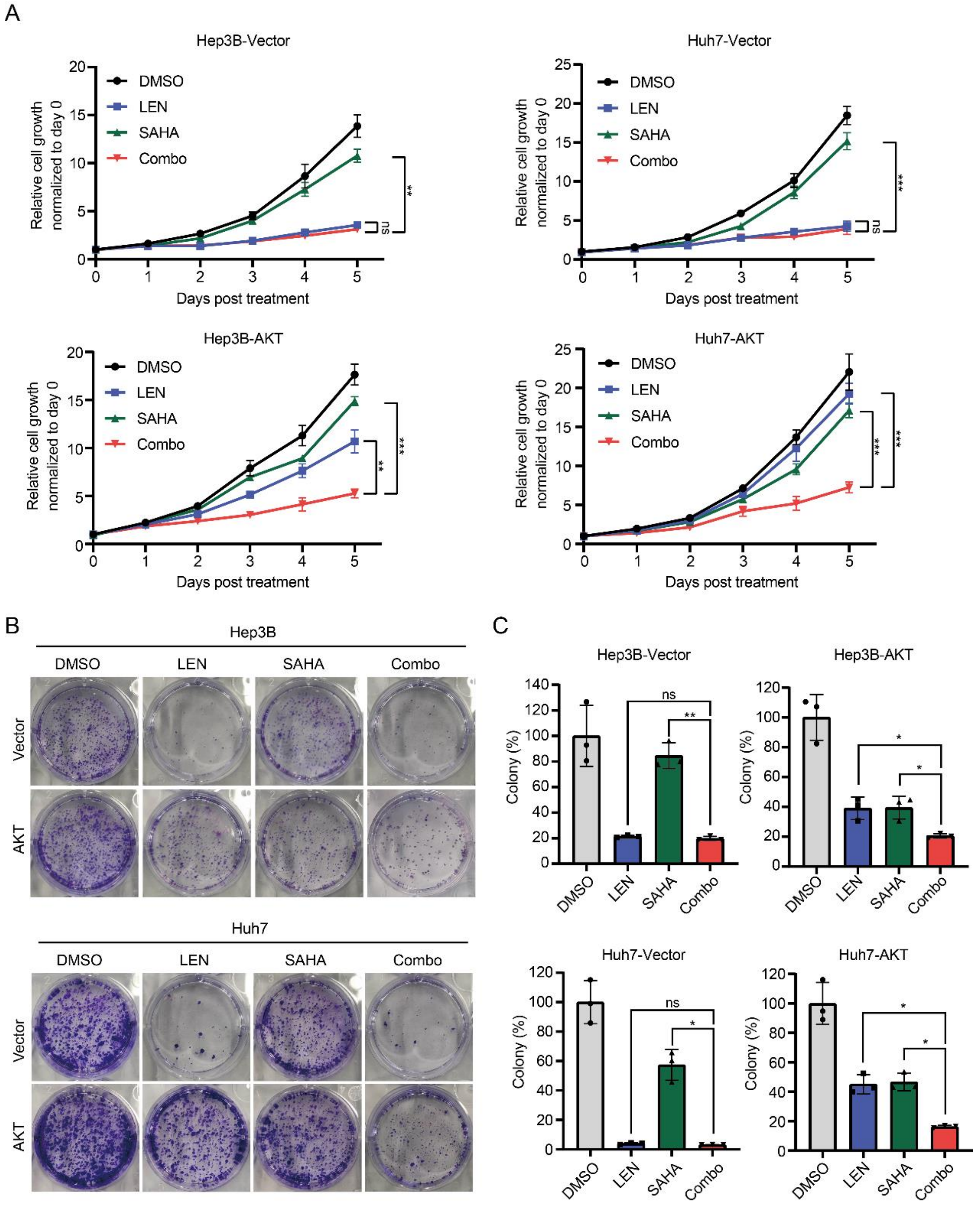
A
Hep3B-Vector
Huh7-Vector
Hep3B-AKT
Huh7-AKT
DMSO
LEN
SAHA
Combo
Relative cell growth normalized to day 0
Days post treatment
ns
**
***
B
Hep3B
Huh7
Vector
AKT
C
Colony (%)

Figure S5

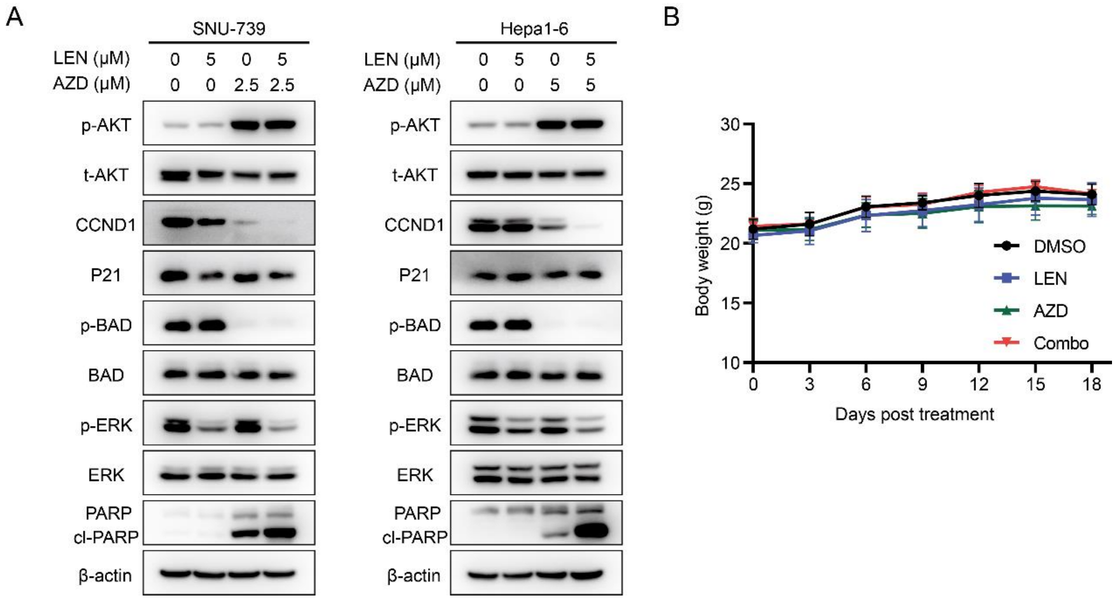
A
SNU-739
LEN (μM) 0 5 0 5
AZD (μM) 0 0 2.5 2.5
Hepa1-6
LEN (μM) 0 5 0 5
AZD (μM) 0 0 5 5
p-AKT
t-AKT
CCND1
P21
p-BAD
BAD
p-ERK
ERK
PARP
cl-PARP
β-actin
B
Body weight (g)
Days post treatment
DMSO
LEN
AZD
Combo

Figure S6

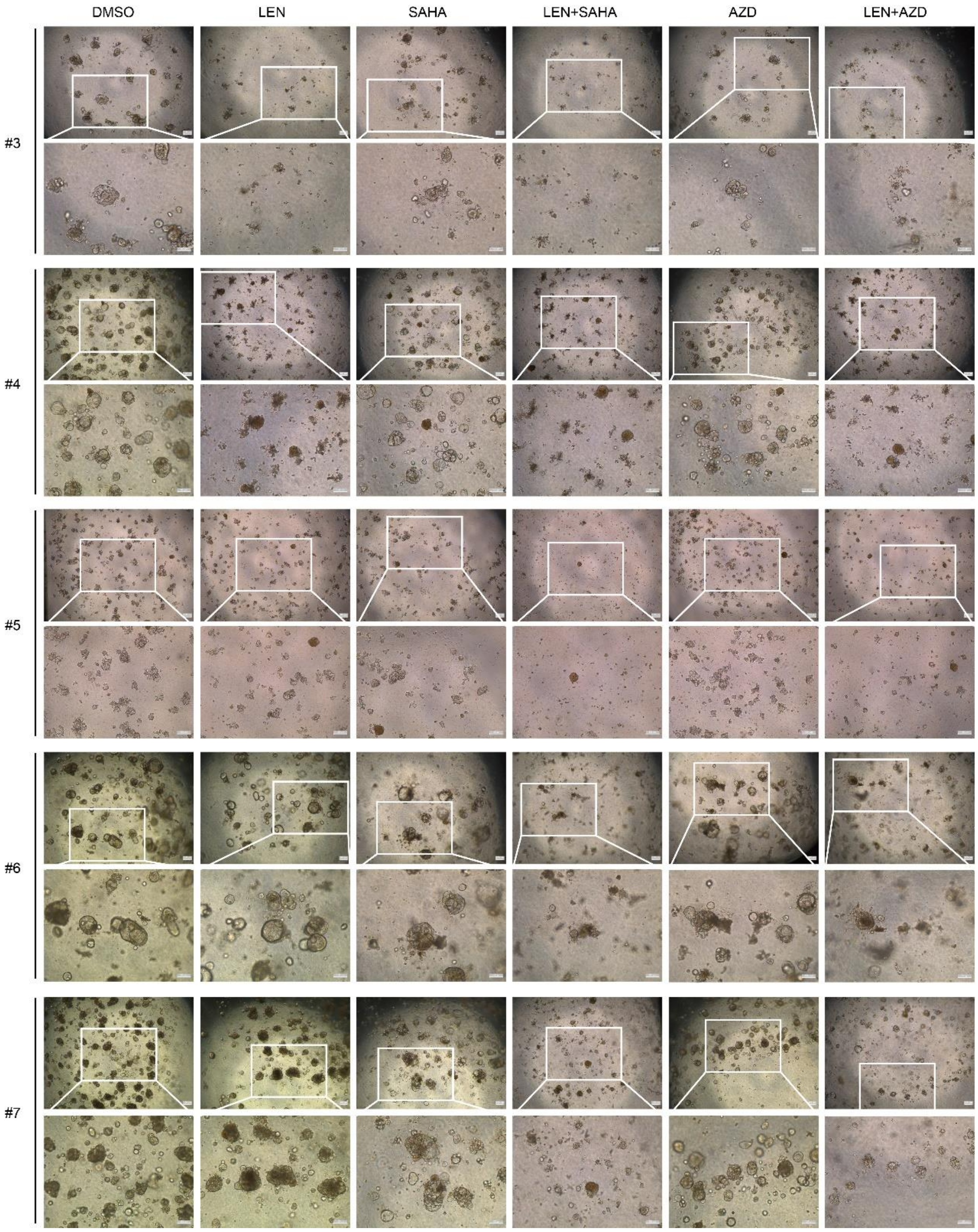

Figure S7

Supplementary Tables

Table S1. Interfering sequences against AKT and HDACs.

siAKT-1 (Human)	5′-CCAUGAAGAUCCUCAAGAATT-3′
siAKT-2 (Human)	5′-UGCCCUUCUACAACCAGGATT-3′
siAKT-3 (Human)	5′-GGACAGAGGAGCAAGGUUUTT-3′
siAkt-1 (Mouse)	5′-GCACAUCAAGAUAACGGACTT-3′
siAkt-2 (Mouse)	5′-GCACCUUUAUUGGCUACAATT-3′
siAkt-3 (Mouse)	5′-GGUAUUUCGAUGAGGAGUUTT-3′
siHDAC1-1 (Human)	5′-GCCGGUCAUGUCCAAAGUATT-3′
siHDAC1-2 (Human)	5′-UACUUUGGACAUGACCGGCTT-3′
siHDAC1-3 (Human)	5′-GCUUCAAUCUAACUAUCAATT-3′
siHDAC2-1 (Human)	5′-UCAUCAGAGAGUCUUAUAUAUTT-3′
siHDAC2-2 (Human)	5′-CCGUAAUGUUGCUCGAUGUTT-3′
siHDAC2-3 (Human)	5′-GACAAACCAGAACACUCCAGAAUAUTT-3′
siHDAC3-1 (Human)	5′-GAGCAACCCAGCUGAACAATT-3′
siHDAC3-2 (Human)	5′-GCCGGUUAUCAACCAGGUATT-3′
siHDAC3-3 (Human)	5′-UAGAGGGAUAUUGAAGCUCTT-3′
siHDAC4-1 (Human)	5′-AAAUUACGGUCCAGGCUAATT-3′
siHDAC4-2 (Human)	5′-AAGUCUGAGCCUCGAUCAGTT-3′
siHDAC4-3 (Human)	5′-GGAAUCUGAACCACUGCAUTT-3′
siHDAC5-1 (Human)	5′-UACGACACGUUCAUGCUAATT-3′
siHDAC5-2 (Human)	5′-AGACGAUAACAGACAGACGTT-3′
siHDAC5-3 (Human)	5′-ACGACACGUUCAUGCUAAATT-3′
siHDAC6-1 (Human)	5′-GCUCGGCCAAGCAAUGGAATT-3′
siHDAC6-2 (Human)	5′-GCAGUUAAAUGAAUUCCAUUGTT-3′
siHDAC6-3 (Human)	5′-GAAACAACCCAGUACAUGAAUTT-3′
siHDAC7-1 (Human)	5′-GGACAAGAGCAAGCGAAGUTT-3′
siHDAC7-2 (Human)	5′-GGCUGGAAACAGAAACCCATT-3′
siHDAC7-3 (Human)	5′-AUCAGUUGCUGCGUCAUGUTT-3′
siHDAC8-1 (Human)	5′-AAGGAUGUACUUAAGACACTT-3′
siHDAC8-2 (Human)	5′-GACGGAAAUUUGAGCGUAUUCUCUTT-3′
siHDAC8-3 (Human)	5′-GGAAUCUGAAGCAUGUGGUCUAGUGACATT-3′
siHDAC9-1 (Human)	5′-CUGAGUAAAUCAGCAACGATT-3′
siHDAC9-2 (Human)	5′-UUUGCUGUCGCAUUUGUUCTT-3′
siHDAC9-3 (Human)	5′-GCUCAAUGCUUCGAAUUCATT-3′
siHDAC10-1 (Human)	5′-GGUUCUGUGUGUUCAACAATT-3′
siHDAC10-2(Human)	5′-GCCGGAUAUCACAUUGGUUTT-3′
siHDAC10-3 (Human)	5′-GCAGGUGAACAGUGGUAUATT-3′
siHDAC11-1 (Human)	5′-GGAGAAGCUGCAUCCCUUUTT-3′
siHDAC11-2(Human)	5′-GGAUGAUGAGUACCUGGAUTT-3′
siHDAC11-3 (Human)	5′-GCAUCAUUGCUGACUCCAUTT-3′

Table S2. Sequences of the DNA primers for qRT-PCR.

PTEN (Human)	Forward	5′-TGGATTCGACTTAGACTTGACCT-3′
	Reverse	5′-GGTGGGTTATGGTCTTCAAAAGG-3′
CDKN1A (Human)	Forward	5′-CGACTGTGATGCGCTAATGG-3′
	Reverse	5′-CCGTTTTCGACCCTGAGAG-3′
CCND1 (Human)	Forward	5′-CTGTGACGCGCAAGTCTGAG-3′
	Reverse	5′-TGCTGGAAACATGCCGGTTAC-3′
AKT1 (Human)	Forward	5′-TGAGCGACGTGGCTATTG-3′
	Reverse	5′-CAGTCTGGATGGCGGTTG-3′
GAPDH (Human)	Forward	5′-GTCTCCTCTGACTTCAACAGCG-3′
	Reverse	5′-ACCACCCTGTTGCTGTAGCCAA-3′
Pten (Mouse)	Forward	5′-TGAAGACCATAACCCACCACAG-3′
	Reverse	5′-ATTACACCAGTCCGTCCCTTTC-3′
Cdkn1a (Mouse)	Forward	5′-TCTTGCACTCTGGTGTCTG-3′
	Reverse	5′-CTGCGCTTGGAGTGATAGAA-3′
Ccnd1 (Mouse)	Forward	5′-AGACCATTCCCTTGACTGC-3′
	Reverse	5′-AAGCAGTTCCATTTGCAGC-3′
Akt1 (Mouse)	Forward	5′-TGTATGAGAAGAAGCTGAGCCC-3′
	Reverse	5′-TCACTGTCCACACACTCCATG-3′
Gapdh (Mouse)	Forward	5′-TGAATACGGCTACAGCAACA-3′
	Reverse	5′-AGGCCCCTCCTGTTATTATG-3′
HDAC1 (Human)	Forward	5′-CATCTCCTCAGCATTGGCTT-3′
	Reverse	5′-CGAATCCGCATGACTCATAA-3′
HDAC2 (Human)	Forward	5′-ATGGCGTACAGTCAAGGAGG-3′
	Reverse	5′-TGCGGATTCTATGAGGCTTCA-3′
HDAC3 (Human)	Forward	5′-CCTGGCATTGACCCATAGCC-3′
	Reverse	5′-CTCTTGGTGAAGCCTTGCATA-3′
HDAC4 (Human)	Forward	5′-GTAAGAAACTTCTAGGCTCGCTC-3′
	Reverse	5′-ACCTCGTTCCATATGGTGTCA-3′
HDAC5 (Human)	Forward	5′-TCTCGGCTCTGCTCAGTGTA-3′
	Reverse	5′-CTGCACACAGCTCCAGTGTT-3′
HDAC6 (Human)	Forward	5′-TGGCTATTGCATGTTCAACCA-3′
	Reverse	5′-GTCGAAGGTGAACTGTGTTCCT-3′
HDAC7 (Human)	Forward	5′-AGAGCAAGCGAAGTGCTGTAG-3′
	Reverse	5′-GGGCTCCAGGGTTCTGTAG-3′
HDAC8 (Human)	Forward	5′-CAGGATGGCATACAAGATGAAA-3′
	Reverse	5′-ATGGGATCCCCAGCTATTGT-3′
HDAC9 (Human)	Forward	5′-ATCCCAAGCTCTGGTACACG-3′
	Reverse	5′-TCTTGTGCTCCTGGTAATGTGT-3′
HDAC10 (Human)	Forward	5′-TGACAACGCCGGATATCACA-3′
	Reverse	5′-CCTCTCCGAACAGCCACATC-3′
HDAC11 (Human)	Forward	5′-GGGTGCCCATCCTTATGGTG-3′
	Reverse	5′-CAGCGGTGTGTCTGAGTTCT-3′

Table S3. Clinicopathological Features of HCC Specimens

Patient number	Gender	Age	Virus	Tumor volume	AFP (ng/ml)	CA199 (U/ml)	CEA (ng/ml)	pT	N	M	Child-pugh	Stage	BCLC stage
#1	Male	69	HBV	7*7.5cm	435.3	56.01	2.16	T3	N0	M0	A	IIIA	B
#2	Male	67	HBV	Diameter 12cm	7.95	71.91	1.23	T1b	N0	M0	A	IB	A
#3	Male	64	HBV	7.5*5cm	631.9	90.33	2.03	T4	N0	M0	B	IIIB	C
#4	Female	74	-	6.2*8.5cm	14.08	69.79	0.48	T1	N0	M0	A	IB	A
#5	Male	70	HBV	11.8*7.7cm	2.45	16.66	3.71	T3a	N0	M0	A	IIIB	B
#6	Male	69	-	2.9*2.2cm	>1210	31.4	3.71	T3a	N0	M0	A	IIIA	B
#7	Male	46	HBV	10.6*6.5cm	>1210	24.2	5.71	T2	N0	M0	A	IIA	A
